# Visual rating versus volumetry of regional brain atrophy and longitudinal changes over a 5‐year period in an elderly population

**DOI:** 10.1002/brb3.1662

**Published:** 2020-05-20

**Authors:** Vilma Velickaite, Daniel Ferreira, Lars Lind, Håkan Ahlström, Lena Kilander, Erik Westman, Elna‐Marie Larsson

**Affiliations:** ^1^ Department of Surgical Sciences, Radiology Uppsala University Uppsala Sweden; ^2^ Division of Clinical Geriatrics Department of Neurobiology, Care sciences and Society Centre for Alzheimer's Research Karolinska Institute Huddinge Sweden; ^3^ Department of Medical Sciences/Clinical Epidemiology Uppsala University Uppsala Sweden; ^4^ Department of Public Health and Caring Sciences Geriatrics Uppsala University Uppsala Sweden

**Keywords:** cognition, dementia, magnetic resonance imaging, neuroimaging

## Abstract

**Introduction:**

The purpose of our study was to compare visual rating and volumetry of brain atrophy in an elderly population over a 5‐year period and compare findings with cognitive test results.

**Materials and Methods:**

Two hundred and one subjects were examined with magnetic resonance imaging (MRI) of the brain. Visual rating and volumetry were performed in all subjects at ages 75 and 80. Cognitive function at both time points was assessed with the Mini‐Mental State Examination (MMSE) and Trail Making Tests A and B (TMT‐A and TMT‐B). Changes in visual rating and volumetry were compared with changes in cognitive test.

**Results:**

A correlation was found between visual rating of medial temporal lobe atrophy (MTA) and hippocampal volumetry at both time points (rs = −.42 and rs = −.49, *p* < .001, respectively). The correlation between visual rating of posterior atrophy (PA); frontal atrophy (F‐GCA) and volumetry of these brain regions was significant only at age 80 (rs = −.16, *p* = .02 for PA and rpb = .19, *p* = .006 for F‐GCA). Visual rating showed only a minimal progression of regional atrophy at age 80, whereas volumetry showed 2%–5% decrease in volume depending on brain region. Performance in the MMSE, TMT‐A, and TMT‐B was virtually unchanged between ages 75 and 80.

**Conclusion:**

We found a mild age‐associated decrease in regional brain volumes in this healthy cohort with well‐preserved cognitive functions. Visual assessment may not be sufficient for detecting mild progression of brain atrophy due to normal aging, whereas volumetry is more sensitive to capture these subtle changes.

## Graphical abstract

Purpose of our study was to compare visual rating and volumetry of brain atrophy in an elderly population over a 5‐year period and compare findings with cognitive test results. We found a mild age‐associated decrease in regional brain volumes in this healthy cohort with well‐preserved cognitive functions. Visual assessment may not be sufficient for detecting mild progression of brain atrophy due to normal aging, whereas volumetry is more sensitive to capture these subtle changes.

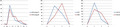

## 1 INTRODUCTION

Structural magnetic resonance imaging (MRI) has become an essential tool in the clinical investigation of patients with neurodegenerative disorders and in clinical trials investigating response to treatment. Volume measurements of various brain regions, based on MRI, are increasingly being used to assess neurodegeneration in Alzheimer's disease (AD) and other cognitive disorders (Huppertz, Kroll‐Seger, Kloppel, Ganz, & Kassubek, 2010).

Different brain areas have different aging trajectories, and normally aged brains undergo changes in volume in the absence of neurodegenerative diseases (Fjell, Walhovd, et al., 2009; Jack et al., 1998; Raz et al., 2005; Resnick, Pham, Kraut, Zonderman, & Davatzikos, 2003). Age‐related brain atrophy of the hippocampi and frontal regions has been extensively documented, while other regions, such as the parietal or occipital lobes, are usually less affected (Fjell, Walhovd, et al., 2009; Jack et al., 1998; Raz et al., 2005; Resnick et al., 2003). Atrophy of different regions of the brain can be assessed visually, using established rating scales, or measuring volume of different brain regions, using automated software packages.

Examination of a healthy cohort helps to understand healthy aging and provides a basis for comparison with pathological conditions.

As the brain ages, changes in cognition are also noticed. Some studies document links between regional changes in the brain and specific cognitive domains, such as between hippocampi and memory or between loss of gray and white matter and deterioration in executive functions (Chen, Chuah, Sim, & Chee, 2010; Ystad et al., 2009). Reduction in specific cognitive domains is also seen in healthy aging (Fjell & Walhovd, 2010).

Numerous cross‐sectional imaging studies have found a correlation between increasing age and decreasing brain volume (Coffey et al., 1992; Good et al., 2001; Mueller et al., 1998; Resnick et al., 2000). Although cross‐sectional studies can generate data quickly, they do not allow for the investigation of individual effects. Longitudinal studies can help avoid some of the problems due to inter‐individual variation. Also, longitudinal data allow for a better understanding of the changes in brain volume and cognition over time. The longitudinal studies published to date show divergent results regarding changes in brain volume and cognition, which partially can be explained by short follow‐up times or inclusion of relatively small cohorts (Leong et al., 2017; Scahill et al., 2003; Tang, Whitman, Lopez, & Baloh, 2001). Also, many studies aim at predicting development of dementia and focus on selected subjects.

In this cohort of elderly, mainly healthy subjects, who were 75 years old at baseline, we investigated the parenchymal reduction in several brain regions (the medial temporal, frontal, and parietal lobes) over a 5‐year period. We compared changes in visual rating of atrophy with changes in volume of corresponding brain regions over time to determine whether volumetry is more sensitive to subtle changes than visual rating for this purpose. We also investigated whether changes in brain morphology in our population correlated with changes in cognitive state.

## 2 MATERIALS AND METHODS

### 2.1 Participants

The Prospective Investigation of the Vasculature in Uppsala Seniors (PIVUS) is a population‐based study of 1,016 subjects, recruited at age 70 years. Subjects were included randomly from the population register of the municipality. Medical history (myocardial infarct, stroke, angina pectoris, diabetes, and other conditions) and information on drug usage (antihypertensive or other cardiovascular medication, diuretics, statins, oral antidiabetics, etc.) were based on self‐reporting. The presence of previous or current diseases was not an exclusion criterion.

Magnetic resonance imaging of the brain was undertaken in 406 of these subjects (randomly selected) 5 years later, at age 75.

Cognitive evaluation of subjects at age 75 was done with the Mini‐Mental State Examination (MMSE), and the Trail Making Test A and B (TMT‐A and TMT‐B) (Folstein, Folstein, & McHugh, 1975; Salthouse, 2011).

An MRI of the brain was repeated in 250 subjects who returned for a 5‐year follow‐up examination at age 80. Cognitive evaluation of subjects at this age was repeated using the MMSE, TMT‐A, and TMT‐B. Changes in results of cognitive tests were calculated by extracting test results at 80 years from test results at 75 years for MMSE and subtracting test results at 75 years from test results at 80 years for TMT‐A and TMT‐B tests.

All medical records were screened in order to identify subjects with diagnosed mild cognitive impairment or dementia disorders at baseline and follow‐up.

The local ethics committee approved the study, and all subjects provided written informed consent.

### 2.2 MRI and visual ratings of regional brain atrophy

Magnetic resonance imaging of the brain was performed with a 1.5 T MR scanner (Intera; Philips Healthcare). A sagittal T1‐weighted 3D gradient echo sequence (echo time 4.0 ms, repetition time 8.6 ms, flip angle 8°, resolution 0.94 × 0.94 × 1.2 mm and matrix 256 × 256 × 170) was interactively reconstructed to 1.2‐mm thick images in a Picture Archiving and Communication System (VuePACS, Carestream; Carestream Health, Inc.) for visual assessment.

An experienced neuroradiologist (VV) performed the visual ratings. For medial temporal lobe atrophy (MTA) scoring, the Scheltens' scale was used (Scheltens et al., 1992). Right and left sides were evaluated separately and average grades for both sides were calculated. For posterior atrophy (PA), the Koedam's scale was used, and for frontal lobe atrophy (F‐GCA), the frontal subscale of Pasquier's scale for global cortical atrophy was used (Koedam et al., 2011; Pasquier et al., 1996). Scans performed at age 75 were rated first. Then, the images obtained in the 80‐year‐olds were rated together with images and visual ratings obtained at age 75 for comparison, like in a clinical context. Changes in atrophy grade were calculated by subtracting grade at 75 years from grade at 80 years old.

### 2.3 Volumetric analysis

Volumetric analysis was performed with FreeSurfer (version 6.0.0), which is a freely available software package for the analysis and visualization of structural and functional neuroimaging data. FreeSurfer provides completely automated parcellation of the brain cortex as well as automated segmentation of subcortical structures.

All raw T1‐weighted DICOM data images were uploaded to the FreeSurfer program, which processed and analyzed the data (Fischl, 2012; FreeSurfer, 2013). The FreeSurfer longitudinal stream was used to extract regional cortical thickness and subcortical volumetric measures and included several steps described in detail elsewhere (Falahati et al., 2017). All output images were inspected for errors by an experienced neuroradiologist (VV). The output of volumetric analysis included volumes of different brain regions including the hippocampi on the right and left sides separately. All volumes selected for further analysis were normalized by dividing the region of interest by the total intracranial volume estimation, also obtained from FreeSurfer. The volumes of frontal and parietal lobes were determined by summation of values of regions as described in the FreeSurfer homepage (FreeSurfer, 2013). In brief, frontal lobe volumes included superior frontal gyrus, rostral and caudal middle frontal gyri, pars opercularis, pars triangularis, and pars orbitalis of the inferior frontal gyrus, lateral and medial orbitofrontal gyri, precentral, and paracentral gyri and frontal pole. The parietal lobe volumes included the superior and inferior parietal, supramarginal, postcentral gyri, and precuneus (Desikan et al., 2006).

Data were processed through the Hive database system (theHiveDB) (Muehlboeck, Westman, & Simmons, 2014).

Changes in volume were calculated by extracting volume of examined lobe at 80 years from volume at 75 years.

### 2.4 Statistics

Statistical analysis was performed with the SPSS software (v.25; IBM). Spearman's rank and point‐biserial correlations were used to investigate associations between variables. Results were interpreted according to the following criteria: .00–.19 means “very weak” correlation, .20–.39 “weak” correlation, .40–.59 “moderate” correlation, .60–.79 “strong” correlation and .80–1.0 “very strong” correlation (Myers, 2003).

Correlation results were considered significant when *p* ≤ .05 (two‐tailed).

Wilcoxon test and the paired *t*‐test were used to analyze changes in atrophy ratings and volume over the time. Correlations were also used to test the association between changes in atrophy rating and volumetry with changes in cognitive test results over 5 years.

## 3 RESULTS

In this study, we used data that included visual ratings of atrophy, volumetric analysis of all MRI scans with sufficient image quality, and cognitive tests in a cohort of subjects at ages 75 and 80. The final sample consisted of 201 subjects, 94 women and 107 men. At age 75, none of the participants had dementia or mild cognitive impairment (MCI). After 5 years, only six of them had developed mild cognitive impairment or dementia: MCI in two, AD in two, and unspecified dementia in two. The cohort is described in Table 1.

**TABLE 1:** Descriptive data of the study cohort (*N* = 201)

	75 years	80 years
Gender, women/men, %	47/53	47/53
Dementia or MCI, *N*	0	6
MMSE, mean ± *SD* (points)	29 ± 1	28 ± 2
TMT‐A, mean ± *SD* (s)	53 ± 17	59 ± 32
TMT‐B, mean ± *SD* (s)	140 ± 86	146 ± 75
MTA average, median	1	2
PA, median	1	2
F‐GCA, median	1	1
HC average, mean ± *SD*	22.6 ± 2.5	21.5 ± 2.7
PL, mean ± *SD*	655.4 ± 48.9	638.9 ± 50.8
FL, mean ± *SD*	988.3 ± 69.7	972.7 ± 73.3

Abbreviations: F‐GCA, frontal lobe atrophy; FL, frontal lobe volume; HC, hippocampus volume; MCI, mild cognitive impairment; MMSE, Mini‐Mental‐State‐Examination; MTA, medial temporal lobe atrophy; *N*, number; PA, posterior atrophy; PL, parietal lobe volume; TMT‐A, Trail Making Test A; TMT‐B‐Trail, Making Test B.

### 3.1 Visual atrophy rating and volumetry at baseline and after 5 years

**FIGURE 1:**
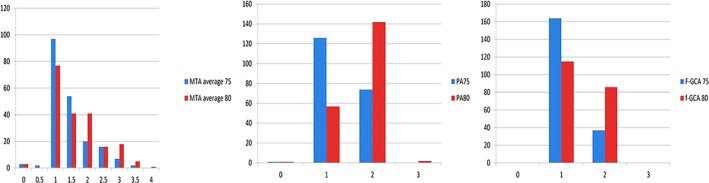
Distribution of atrophy grades (MTA average, PA, and F‐GCA) at age 75 (a) and at age 80 (b). F‐GCA, frontal lobe atrophy; MTA, medial temporal lobe atrophy; PA, posterior atrophy. Values in the *x*‐axis show atrophy grades (MTA 0–4, PA, and F‐GCA 0–3). Values in the *y*‐axis show number of subjects

The distribution of atrophy scores at age 75 and at age 80 in the whole cohort is presented in Figure 1. The scores for each individual are the average of the left and right‐sided scores.

At age 75, the majority of subjects (48%) had an average MTA grade 1 (97 out of 201), followed by grade 1.5 (27% or 54 out of 201). Only three subjects had an average MTA grade 0, and none had grade 4 as average MTA. The median for MTA at age 75 was 1. After 5 years, the majority (38%) still had an average MTA grade 1 (77 out of 201), followed by grades 1.5 and 2 (both 20% and 41 out of 201). The number of subjects with MTA grade 0 remained the same, and only one subject had an average MTA grade 4. The median grade increased to 2 after 5 years.

At age 75, none had PA grade 3, and only one individual had PA grade 0. The majority (63%; 126 out of 201) had PA grade 1, and the rest (almost 37%) had PA grade 2. After 5 years, two subjects had PA grade 3, and the majority (71%; 142 out of 201) had PA grade 2. The median for PA increased from 1 at 75 years to 2 at 80 years.

As for F‐GCA, at the age of 75, none had atrophy grade 0 or 3, and the majority (82%; 164 out of 201) had atrophy grade 1, while 18% of the subjects had grade 2. After 5 years, still none had atrophy grade 3 in the frontal lobes, but the number of subjects with grade 1 F‐GCA decreased to 115 (57%), and those with grade 2 F‐GCA increased to 37 (43%).

The median for F‐GCA was 1 both at 75 and 80 years.

Changes in visual ratings of atrophy were examined using the Wilcoxon test, which showed that after 5 years the shift toward a higher grade of atrophy in all brain regions was small, but statistically significant (*p* < .001).

**FIGURE 2:**
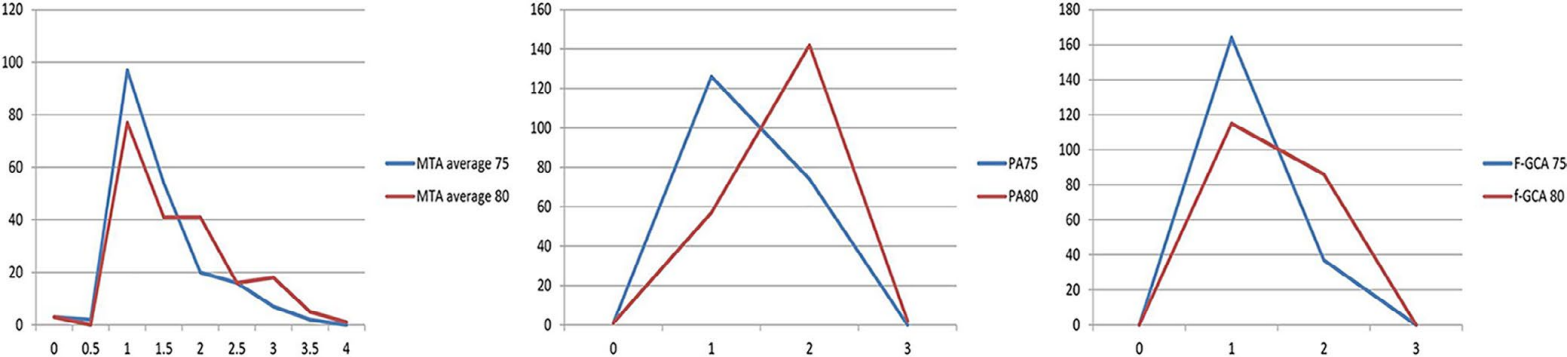
Changes in grades of MTA average, PA, and F‐GCA at age 75 and at age 80. F‐GCA, frontal lobe atrophy; MTA, medial temporal lobe atrophy; PA, posterior atrophy. Values in the *x*‐axis show atrophy grades (MTA 0–4, PA, and F‐GCA 0–3). Values in the *y*‐axis show number of subjects

Changes in visual ratings of regional brain atrophy after 5 years in the whole cohort are shown in Figure 2.

Among the six subjects who later developed MCI or dementia, only one had MTA grade 0 and one grade 1; the other subjects had MTA grade 2 and grade 3, in equal distribution. None of them had MTA grade 4 at age 75. As for PA, four of these subjects had PA grade 1, the other two PA grade 2. All these six subjects had F‐GCA grade 1 at age 75.

Volumetric analysis showed that average hippocampal volume decreased by 5% over 5 years in the entire cohort. The volume of the parietal lobes, decreased by 3% over 5 years, and the volume of the frontal lobes decreased the least of all, only by 2% over 5 years.

All changes in volumetry, evaluated using the paired *t*‐test, were statistically significant (*p* < .001).

The average decrease in total hippocampal volume in those six subjects who developed MCI or dementia was 17% over 5 years. The volume of the parietal lobes in these subjects decreased 6% and the volume of the frontal lobes 4% during the course of 5 years.

### 3.2 Visual ratings versus volumetry

Visual ratings of atrophy were compared with volumetric analysis performed with the FreeSurfer software in subjects at age 75 and age 80.

Visual ratings of MTA at age 75 correlated statistically significantly with hippocampal volumetry, although the correlation was moderate (rs = −. 42, *p* < .001; Table 2). At age 80, the correlation between visual rating of MTA and hippocampal volumetry was still moderate (rs = −.49, *p* = .001; Table 2).

**TABLE 2:** Correlation between visual rating of atrophy and volumetry at ages 75 and 80

	At 75 years	At 80 years
MTA average versus HC average	rs = −.42, *p* < .001	rs = −.49, *p* < .001
PA versus PL	rpb = .07, *p* = .316	rs = −.16, *p* = .02
F‐GCA versus FL	rpb = .03, *p* = .724	rpb = .19, *p* = .006

Abbreviations: F‐GCA, frontal lobe atrophy; FL, frontal lobe volumetry; HC, hippocampal volumetry; MTA, medial temporal lobe atrophy; PA, posterior atrophy; PL, parietal lobe volumetry; rpb, point‐biserial correlation coefficient; rs, Spearman's correlation coefficient.

In the visual rating of PA, none of the participants had a score of 3, and only one individual had a score of 0, the rest had scores of 1 or 2. Dummy variables were created and statistical calculations were performed with point‐biserial correlation. There was no significant correlation between the visual ratings of PA and volumetry of parietal lobes at age 75 (*p* = .316; Table 2). At age 80, a few subjects already had PA grade 3, so, statistical calculations were performed with Spearman's test. This time, a statistically significant correlation was found (rs = −.16, *p* = .020).

As mentioned above, none of the subjects had a score of 0 or 3 in the visual rating of F‐GCA at either age 75 or age 80. Thus, since only two scores were present (1 and 2), dummy variables in both cases were created. Statistically significant correlation was found in 80‐year‐old subjects (rpb = .19, *p* = .006), but not in 75‐year‐olds. All the results are presented in Table 2.

### 3.3 Association between visual ratings, volumetry, and cognition

None of the subjects had any cognitive impairment at the age of 75. After 5 years, only six subjects out of 201 (3%) developed MCI or dementia. Further, results in all cognitive tests were virtually unchanged (Table 1). In the six subjects who developed MCI or dementia, changes in cognition were more pronounced: the MMSE results decreased from 28 ± 1.7 to 23 ± 4.3 points; and the time to complete the TMT‐A increased from 72 ± 21.3 to 85 ± 35 s and the TMT‐B from 134 ± 62 to 152 ± 200 s.

At baseline, statistically significant correlations were found only between the average MTA and MMSE (*p* = .015). Statistically significant correlations were found between the average hippocampal volume and MMSE and TMT‐B tests (*p* = .013 and *p* = .006). No statistically significant correlation was found between visual rating or volumetry of PA or F‐GCA and any cognitive tests.

At age 80, a statistically significant correlation was found between the MMSE, TMT‐B tests, and average MTA (*p* = .003 and *p* = .001, respectively) and between the MME, TMT‐B test, and average hippocampal volume (*p* < .01). Neither average MTA or average hippocampal volume correlated with TMT‐A test. Again, no statistically significant correlation was found between visual rating or volumetry of PA and any cognitive tests. This time, statistically significant correlations were found between the TMT‐B test and visual rating of frontal atrophy and volumetry (*p* = .001 and *p* = .024, respectively).

When we analyzed correlations between changes in visual ratings, volumetry of different lobes and changes in cognitive tests, statistically significant correlations were found between changes in averaged hippocampus volumetry and MMSE (*p* = .027), changes in volume of parietal lobe and changes in MMSE, and TMT‐B (*p* = .012 and *p* = .011, respectively), and between changes in volumetry of frontal lobes with changes in MMSE and TMT‐A ( *p* = .027 and *p* = .002, respectively). No correlation was found between changes in atrophy, which was not more than 1 grade, as measured by visual rating, and changes in any of cognitive tests. All results are presented in Table 3.

**TABLE 3:** Correlation between changes in atrophy ratings and volumetry with changes in cognitive tests

	MMSE change	TMT‐A change	TMT‐B change
MTA change	*r* = .086, *p* = .237	*r* = .096, *p* = .19	*r* = .136, *p* = .066
PA change	rpb = −.099, *p* = .137	rpb = −.074, *p* = .311	rpb = −.05, *p* = .5
F‐GCA change	rpb = −.008, *p* = .913	rpb = −.034, *p* = .638	rpb = .077, *p* = .302
HC change	*r* = .16, *p* = .027	*r* = .005, *p* = .947	*r* = .041, *p* = .579
PL change	*r* = .181, *p* = .012	*r* = −.122, *p* = .094	*r* = .186, *p* = .011
F‐GCA change	*r* = .16, *p* = .027	*r* = −.222, *p* = .002	*r* = .1, *p* = .177

Abbreviations: F‐GCA, frontal lobe atrophy; FL, frontal lobe volumetry; HC, hippocampal volumetry; MTA, medial temporal lobe atrophy; PA, posterior atrophy; PL, parietal lobe volumetry; *r*, Pearson's correlation coefficient; rpb, point‐biserial correlation coefficient.

## 4 DISCUSSION

The present study investigated changes in visual rating and volumetry of regional brain atrophy and cognitive function from age 75 to age 80 in a population‐based cohort, where all individuals were cognitively unimpaired at baseline and only six progressed to MCI or dementia at age 80. Our study showed that in elderly adults, longitudinal changes in brain volume, measured by both visual rating and volumetry, are not uniform (the magnitude of change varies across regions). On average, the degree of atrophy measured by visual rating of MTA, PA, and F‐GCA increased by <1 grade, while volumetry showed 5%, 3%, and 2% volume loss in these regions. The greatest changes were observed in the temporal regions and were less pronounced in the parietal and frontal lobes.

There were only minimal changes in visual ratings of atrophy in different brain regions in the form of slight increases in atrophy grade, mostly in MTA, less in PA, and even less in F‐GCA. The median value for MTA increased from grade 1 to grade 2. Two recent studies showed that an MTA grade 2 is considered abnormal at ages 75 and 80 for the discrimination between cognitively unimpaired subjects and patients with AD (Claus et al., 2017; Ferreira et al., 2015). The fact that the MTA grade 1 was the most common grade in our study at age 75, when all subjects were cognitively unimpaired, but the MTA grade 2 was the most common grade at age 80, when several subjects progressed to MCI or dementia, may support the MTA grade 2 cut‐off for discriminating cognitive impairment. Those two studies were cross‐sectional and, a novelty of our study is that, to our knowledge, no previous publication has evaluated the change of visual scoring grades over time. Further, these two previous studies were based on patients with diagnosed AD or MCI, or included both patients with diagnosed dementia and control subjects, while our study is population‐based, with cognitively intact subjects at baseline and six subjects developing MCI or dementia at follow‐up.

Posterior atrophy increased with the same value as MTA, from median grade 1 to grade 2. One of the above‐mentioned studies showed that the cutoff value for PA at both age 75 and age 80 should be grade 1, whereas our study showed that PA grade 2 is common in a cohort of mostly cognitively unimpaired subjects. This difference may be explained by differences in the populations (the ADNI cohort, which was used in the previous study, included amnestic patients with prominent pathology in medial temporal lobes while posterior cortex could be less affected) or in the visual ratings per se. The grade of F‐GCA was the same at both time points, median 1. The above‐mentioned study (Ferreira et al., 2015) considered F‐GCA grade 1 as the abnormal cutoff to discriminate between cognitively unimpaired subjects and AD patients both at age 75 and 80. Therefore, as for PA, our study revealed higher degrees of atrophy in a population‐based cohort. Although the published cutoffs showed good performance in the ADNI cohort and were validated in several clinical setting cohorts (Ferreira et al., 2015; Ferreira, Jelic, et al., 2016), future studies are thus warranted to validate these cutoffs of visual rating scales at the population level.

The hippocampal volume as quantified with FreeSurfer decreased by 5% after 5 years, on average 1% per year, which is in agreement with previous studies, both cross‐sectional and longitudinal (Jack et al., 1998; Scahill et al., 2003). Loss of hippocampal volume in the six subjects who after 5 years developed MCI or dementia was more severe: on average 17% over 5 years, or 3.4% per year. This is less than in other published studies (Cavallin, Bronge, et al., 2012; Jack et al., 1998). The explanation for this discrepancy may be that we examined a population‐based cohort, not one from a memory clinic, and that there was a very small number of subjects who progressed to MCI or dementia in our cohort (Jack et al., 1998). The volume of the parietal lobes decreased by 3% and the volume of the frontal lobes by 2% over 5 years (0.6%–0.4% per 1 year). Some earlier studies based on subjects of various ages showed more prominent loss of gray matter with age (Kruggel, 2006; Tang et al., 2001). Other studies showed volume rate loss (up to 0.32%–0.5%) similar to that found in our study (Chan et al., 2001; Jack et al., 2005; Mueller et al., 1998; Scahill et al., 2003). According to numerous studies, loss of cortex volume in frontal regions in healthy nondemented subjects is usually greater than in temporal or parietal regions (Driscoll et al., 2009; Fjell & Walhovd, 2010; Fjell, Westlye, et al., 2009; Jack et al., 2005; Leong et al., 2017; Raz et al., 2005; Resnick et al., 2003). Other studies showed more effects of age on the parietal cortex (Brickman, Habeck, Zarahn, Flynn, & Stern, 2007; Fjell, Westlye, et al., 2009; Good et al., 2001; Resnick et al., 2003; Salat et al., 2004). Our study, however, showed greater loss of volume in the hippocampal region than in the frontal or parietal lobes. The literature is thus varied, and the differences can probably be explained by variation in the age range of the examined cohort and the measurements of examined regions.

The correlation between visual scoring of atrophy and volumetry at age 75 was found with regard to only MTA and hippocampal volumetry. Similar results have been reported before, although with correlations of various strengths. For example, some studies showed a correlation strength similar to our study (Boutet et al., 2012; Cavallin, Bronge, et al., 2012; Persson et al., 2018). Another study, although cross‐sectional, showed a much stronger correlation between MTA and hippocampal volumetry (−0.753 to −0.767) (Persson et al., 2018).

At the age of 80, the correlation between MTA and hippocampal volumetry increased, and significant correlations, although very weak, were found between PA and parietal lobe volumetry and between F‐GCA and frontal lobe volumetry. Results in other studies showed better correlations than in our study, especially for the frontal region (Driscoll et al., 2009; Ferreira, Cavallin, et al., 2016). Also, one study showed that atrophy in the frontal regions can be more visible only at latter follow‐up examination (Raz, Ghisletta, Rodrigue, Kennedy, & Lindenberger, 2010). These discrepant results in relation to our study can probably be explained by a very limited variation of atrophy grades in our visual ratings, showing that the human eye is probably less sensitive in detecting small changes. It also supports the theory that machine‐based analysis is more sensitive to subtle progressive changes in brain atrophy than the human eye, both in cross‐sectional and longitudinal studies (Ross et al., 2015; Ross, Ochs, Seabaugh, Shrader, & Neuroimaging, 2013; Tuokkola et al., 2016, 2018).

Our cohort maintained relatively normal cognitive status after 5 years, measured with the MMSE and the TMT test. Correlations between visual ratings of atrophy and cognitive tests were minimal and significant only at age 80. Our results agree with the results described in our earlier publication for age 75 (Velickaite et al., 2018). One of the reasons for such minimal changes could be that in the current study we examined a cognitively healthy population in which none had dementia or MCI at the age of 75. In this cohort, average performance in the tests showed no significant deterioration at follow‐up.

In the whole cohort, analyzing changes in atrophy in association with changes in cognitive tests over 5 years, we found no significant association between development of atrophy in the examined lobes (as assessed through visual rating) and changes in any cognitive tests. Change in average volume of hippocampi correlated significantly only with changes in MMSE, which agrees with other studies (Fjell, Amlien, Westlye, & Walhovd, 2009; Yamaguchi et al., 2002).

Change in volume of parietal lobes correlated significantly with changes in MMSE and TMT‐B test. Change in frontal lobe volume also correlated with changes in MMSE and TMT‐A test. This is partly in agreement with the double dissociation between AD and healthy aging theory, which stipulates that early AD affects the medial temporal lobes, whereas an anterior to posterior atrophy is seen in healthy aging and is associated with executive problems (Head, Snyder, Girton, Morris, & Buckner, 2005).

More severe cognitive decline in the subjects who developed MCI or dementia was associated with greater atrophy in all brain regions. This is in line with studies reporting that cognitive decline is often associated with more marked atrophy (Brickman et al., 2007; Leong et al., 2017). Nonetheless, we believe that in our study, the very small number of subjects who developed MCI or dementia should not significantly affect the results of the whole cohort.

Longitudinal studies have several challenges. First, changes in the hardware and software of the scanner can affect the measurements. In this study, all subjects were examined with the same MRI scanner at both time points, with some inevitable routine software upgrades between examinations, which is unlikely to bias our findings. Also, drop‐out rates from a cohort can influence the final results. Here, we analyzed the same participants at two time points. Longitudinal studies are important in determining the clinical relevance of observed brain changes and their relation with cognitive decline. In scientific studies with longitudinal comparisons, automated methods like volumetry would be preferable, since human raters have different overtime reliabilities (Cavallin, Loken, et al., 2012).

In conclusion, our results indicate that a majority of cognitively healthy elderly subjects at age 75 have only very slight increase of atrophy grade over 5 years as judged by visual rating. These small changes can be captured better with volumetry, which is more sensitive than visual rating. Also, the subjects in this study experienced only minimal decline of cognition between age 75 and age 80, and most of these changes correlated significantly with the changes in brain volume. Therefore, volumetry can be more advisable to follow loss of brain volume in cognitive healthy individuals.

## CONFLICT OF INTEREST

None.

## AUTHOR CONTRIBUTION

All authors made a substantial contribution to the conception, design, and revision of the work. VV collected the data, performed visual rating, volumetry of brain regions, the statistical analysis, and wrote the first draft. LK examined cognitive status of all the patients. DF provided consultations in statistical analysis. EML, EW, LL, and HÅ supervised the work, provided consultations, and revised the manuscript. All authors were involved in the final approval of the manuscript.

## Data Availability

Research data are not shared.
